# Cognitive and Emotional Perceptions of Illness in Patients Diagnosed with Type 2 Diabetes Mellitus

**DOI:** 10.3390/healthcare12020199

**Published:** 2024-01-15

**Authors:** Lucija Gosak, Gregor Stiglic

**Affiliations:** 1 Faculty of Health Sciences, University of Maribor, 2000 Maribor, Slovenia; gregor.stiglic@um.si; 2 Faculty of Electrical Engineering and Computer Science, University of Maribor, 2000 Maribor, Slovenia; 3 Usher Institute, University of Edinburgh, Edinburgh EH8 9YL, UK

**Keywords:** diabetes type 2 mellitus, illness perception, psychometric properties

## Abstract

Type 2 diabetes mellitus (T2DM) affects a patient’s physical, social, and mental well-being. Perceptions of the illness are linked to quality of life. The aim of this study was to assess illness perception in patients diagnosed with T2DM and to validate the Brief Illness Perception Questionnaire in the Slovenian language. A cross-sectional study involved 141 patients diagnosed with T2DM. We performed a content analysis of the questionnaire and estimated the S-CVI, I-CVI, kappa coefficient. We also used Cronbach’s alpha to assess the reliability. Participants did not have a very threatening perception of T2DM, but being overweight and having cardiovascular disease were significant contributors to a more threatening perception. The most frequently indicated factors influencing the onset and development of T2DM were heredity and genetics, stress and other psychological distress, and poor and inadequate nutrition. I-CVI ranged from 0.833 to 1.00, while the kappa is greater than 0.74, confirming the excellent validity of the questions. The content validity assessment of the questionnaire further confirms that the questionnaire is suitable for use with the target population in Slovenia. The questionnaire proved to be a valid and reliable tool that can be used to assess the relationship between illness perception and self-management of T2DM.

## 1. Introduction

Chronic diseases have an impact on people’s lives, so their management of the situation is a matter of concern [1]. Illness perception involves knowledge about the illness which allows patients to understand their condition [2] and represents acceptance of one’s illness and the possible consequences of the illness [3,4]. Disease perception is an important driver of health [5] as it is important in guiding patients in coping with the illness and in guiding illness-specific behaviours [6], such as self-care, healthcare utilisation and respect for treatment [7].

Illness perception is associated with an individual’s mental health, health behaviours and physical health [4]. Aberkane (2016) found in a study that illness perception does not change with age or deteriorating health [8]. Patients with higher scores often perceive their illness as chronic and therefore have a better understanding of their illness and more control over their treatment [9]. Perceptions of illness are associated with identifying the causative agent, recognising the illness, adhering to the illness, and coping with treatment [8]. Perceptions of illness are more negative when a person has a higher number of illnesses and is more dependent, which in turn affects their quality of life [10]. Nabolsi (2020) states that perceptions of illness do not necessarily correspond to actual illness management [11].

The diagnosis of type 2 diabetes mellitus (T2DM) is a challenge for every patient [12]. The illness affects the physical, social, and mental well-being of people living with it and can have a serious negative impact on the patient’s social life [13]. The aim of treating people with T2DM is to improve their quality of life, avoid complications and reduce additional morbidity and mortality. Their perception of their illness is often intertwined with their quality of life and the state of their illness [14]. In the first few years after being diagnosed with T2DM, patients often do not perceive the illness as serious and delay making changes to their lifestyle until they experience complications [15]. However, negative beliefs about the condition are linked to worse health and outcomes [4]. People with T2DM’s perceptions, beliefs and knowledge influence their level of engagement in T2DM self-care [16,17].

More than half of the patients in the study by Halim et al. (2023) felt that the three most important factors in self-management of the illness were better knowledge about the illness and its treatment, receiving supportive instructions from healthcare providers, and using monitoring devices [18]. Bilondi et al. (2021) concluded that to improve adherence it would be necessary to improve illness awareness through patient education [19]. Patients most often reported that their family and friends and healthcare professionals were the most supportive [1].

The aim of this study was to assess illness perception in patients diagnosed with T2DM and to validate the Brief Illness Perception Questionnaire [20].

## 2. Materials and Methods

### 2.1. Participants

This study included 141 participants diagnosed with T2DM, 59 men (41.8%) and 80 women (56.7%), with two subjects not specifying their gender (1.4%). The mean age of the men was 63.89 (SD = 12.13) years, and the mean age of the women was 62.44 (SD = 13.68) years. The study included people without chronic complications as a result of type 2 diabetes. Most participants were retired (n = 91, 64.5%) or employed (n = 40, 28.4%). A larger proportion of participants were married (n = 89, 63.1%). Most participants had technical, general, or other vocational education (n = 64, 45.4%) and none or primary education (n = 25, 17.7%). The mean time since diagnosis of T2DM was 17.03 years (SD = 15.78).

### 2.2. Research Instrument

The questionnaire Brief Illness Perception Questionnaire from authors Broadbent et al., 2006 [20] provides a tool for rapid assessment of illness perception, which is especially useful in the affected population, large-scale studies, and research plans with multiple measures. The scale measures a patient’s cognitive and emotional perception of their illness including consequences, timeline, personal control, treatment control, identity, compliance, care, emotional response, and causes. The questionnaire contains eight questions, which are evaluated on a scale from 0 to 10 points and one open-ended question. Each item assesses one dimension of illness perception. The total score represents the degree to which the illness is perceived as threatening or beneficial. A higher score reflects a more threatening view of the illness [20]. In patients with kidney disease, the original questionnaire showed good test-retest reliability and concurrent validity with appropriate measures [21].

### 2.3. Data Collection Process

We carried out a cross-sectional study, which was carried out during the second half of 2022 and included patients diagnosed with T2DM. When collecting the data, we considered that we have 10 participants per questionnaire item, which gave us a minimum participant size of 90. Throughout all stages of the research process, we ensured and maintained the principles of anonymity and voluntary participation for all participants. Participants were invited via social media and printed questionnaires were also distributed in health centers to increase the response rate. We included a dual method of collecting responses, as the online survey allows only those who are more digitally literate to be surveyed, while the paper-based format allows those who do not have access to a digital communication tool to be included. It also allowed us to include a more diverse population. The paper-based survey was administered at health centers and participants completed the survey when they were seen by a nurse at the health center. This allowed us to get responses directly from participants and collect data immediately. The questionnaires were given to the participants before the examination so that they could complete them at their leisure and ask for support and further clarification.

We used a Brief Illness Perception Questionnaire, developed by Broadbent et al. in 2006. Before using the questionnaire, we obtained permission from the authors to use the questionnaire in our research. To facilitate comprehension and accessibility for the Slovenian-speaking participants, we conducted back-translation of the Brief Illness Perception Questionnaire [21].

### 2.4. Data Analysis

When illness perception was calculated using the Brief Illness Perception Questionnaire [20], the scores for questions 3, 4 and 7 were reversed and then added to the scores from the remaining questions. All measurements were made on a scale of 0 to 10, except for question 9. Illness perception was assessed based on the following distribution: <42 indicates a low experienced threat, 42–49 indicates a moderate experienced threat and ≥50 indicates a high experienced threat [22]. Question 9 was descriptive and related to the most important causal factor of the T2DM reported by participants. We performed a categorical analysis for the highest reported causal factor, which allowed us to gain more insight into the understanding and classification of illness perceptions [20].

To assess the content validity of the questionnaire, registered nurses with knowledge of T2DM (n = 6) were asked for participation in the study. Their role was crucial in assessing the appropriateness of the content of the questions in the context of their experience. To assess the content validity of the questionnaire, we used the content validity index of individual statements (I-CVI) and the content validity of the whole questionnaire (S-CVI) [23]. We used the I-CVI to assess individual statements, where a value of 0.80 or greater represents a statement that has good content validity [24]. We also calculated the kappa coefficient (κ), which served as an additional indicator to assess the degree of agreement between evaluators [24].

Reliability was measured to determine whether the Brief Illness Perception Questionnaire reliably reflects the same construct that it measures. Cronbach’s alpha, which reflects the internal consistency of the measurement instrument, was used to assess reliability. If Cronbach’s alpha values are between 0.7 and 0.8, this reflects a high degree of internal consistency between the questions of the questionnaire. Some authors also state that values as low as 0.5 are acceptable in the early stages of a survey. Higher Cronbach’s alphas indicate greater internal consistency and therefore measurement reliability [25,26]. In our study, we actively used the central limit theorem (CLT) as the basic statistical tool to report the statistical characteristics of the sample and to validate the hypotheses.

## 3. Results

There was a different percentage of missing values ranging from 12.06% to 13.48% present in data from questions. The mean perception of T2DM score based on Brief IPQ was 32.37 (SD = 10.21) (Table 1), which does not reflect a very threatening view of the illness. Gender has no statistically significant effect on disease perception (*p* = 0.442). A more detailed analysis shows that participants who were obese (n = 58; 41.1%) had a more threatening perception of the illness (33.04 vs. 32.56; *p* = 0.843), compared to participants with a history of cardiovascular disease (n = 21; 14.9%) (34.70 vs. 32.58; 0.463), but there was no statistically significant impact. There was no statistically significant coefficient of correlation between participants’ age and their perception of the illness (*p* = 0.950), suggesting that age per se does not have a significant impact on how participants perceive the severity of T2DM. Duration since diagnosis is positively correlated with disease perception (*p* = 0.028).

Participants in our study identified several key factors that they believed had a significant impact on the onset of T2DM. The most common and prominent factors were heredity and genetics (n = 33), stress and other psychological distress, reflecting recognition of the influence of emotions and psychosocial factors on the illness (n = 24), and poor diet (n = 23). Although less frequently mentioned, obesity (n = 13), age (n = 7), lack of exercise (n = 5), unhealthy lifestyle including a combination of diet, lack of exercise, smoking, alcohol consumption (n = 4) also play an important role. In addition, two participants highlighted the use of medication (n = 2).

Linear regression weakly predicts the dependent variable BIPQ Total Score (*p* = 0.136). Age (*p* = 0.038) and disease duration (*p* = 0.036) significantly contribute to the disease perception of patients with type 2 diabetes (Table 2).

### 3.1. Content Validity of the Questionnaire

To assess the content validity of the illness perception questionnaire by six nurses experienced and knowledgeable in the management of patients with T2DM, a thorough analysis of the nine items of the illness perception questionnaire was carried out. The I-CVI, UA, Pc and k were calculated for each of the 9 items in the questionnaire. The results of our calculations show that the I-CVI indices for all items ranged from 0.833 to 1.00 (Table 3), indicating a high level of agreement between evaluators on the appropriateness of each statement in the questionnaire.

### 3.2. Reliability Analysis of the Questionnaire

The kappa was also greater than 0.74, indicating excellent validity of the questions in the questionnaire (Table 4). The content validity score for the entire questionnaire of 1.000 also confirms that the questionnaire is designed to be appropriate for use by the target population in assessing illness perceptions.

The reliability for the Brief Illness Perception Questionnaire, calculated using Cronbach’s alpha, was 0.652. In our case, although the alpha value does not reach the optimal level, it still indicates a certain degree of internal consistency between the questions in the questionnaire. Further analysis revealed that for the following four specific questions in the questionnaire, the correlation between each item and the total was less than 0.2: ‘How long do you think your illness will last’, ‘How much control do you think you have over your illness’, ‘How much do you think treatment can help your illness’ and ‘How worried are you about your illness’. The lower correlations for these questions may indicate increased variability in participants’ responses.

## 4. Discussion

The results of the survey show that perceptions of T2DM as expressed by the Brief Illness Perception Questionnaire do not reflect a highly threatening attitude towards the disease, with no statistically significant differences observed according to respondents’ gender or age, suggesting that similar patterns of disease perception exist in both men and women. This demonstrates the universality of the factors influencing disease perception. Men have a slightly more threatening perception of the disease than women (5.04 vs. 4.71), but there is no significant difference.

Those who faced additional challenges, such as being overweight or having cardiovascular disease co-diagnosed with type 2 diabetes, were more threatened by their illness. Paz-Salinas et al. (2015) in their study examined perceptions of health in people with diabetes in relation to their BMI. Up to 95% of participants who were overweight or obese considered themselves healthy despite having a diagnosis of diabetes and being overweight or obese. This contrasts with our findings, where participants with T2DM who were overweight had a more threatening view of the illness compared to those of normal weight [27]. Obesity has a significant impact on the development of macrovascular and microvascular complications in T2DM [28], which in turn affects patients’ quality of life and perception of the disease [29]. Sayón-Orea et al. (2018) corroborate our findings, as in their study, participants with normal weight and chronic illness were less likely to report poor health than those with overweight or obesity and chronic disease, particularly if they had been diagnosed with hypertension and T2DM [30].

McCormack & Pamarthi (2019) find that people who are overweight or obese perceive a risk of illness despite inaccurate perceptions of their weight. They also find that people who perceive their illness as serious are more likely to perceive another illness as serious [31]. This is also suggested by our results, as participants who were also diagnosed with cardiovascular disease perceived T2DM as more serious. Similarly, Ogunrinde et al. (2021) found that emotional perceptions of disease are highest in those with at least four different diseases [32].

In our study, we found that participants’ age did not affect how seriously they perceived their illness. These results suggest that age is not an important factor in how people perceive the severity of T2DM. Similarly, Aberkane (2018) found that disease perception did not change with age in older adults with chronic illness [33].

On average, T2DM patients perceive the duration of their disease as chronic (9.02; SD = 2.09). In contrast, Endler et al. (2001) found that participants with acute disease had higher self-efficacy scores compared to those with chronic disease [34].

Heredity and genetics, stress and other psychological distress were among the most reported factors influencing the onset of T2DM in our research. Inadequate and poor diet was another important factor in the occurrence and perception of T2DM among participants. Obesity was mentioned less frequently but was still identified as a factor influencing the development and worsening of the illness. Less frequently mentioned factors that were also considered to have an influence were age, physical inactivity, and an unhealthy lifestyle, which includes a combination of poor diet, physical inactivity, smoking and alcohol consumption. In a study analysing the relationship between T2DM and obesity, Hemanth Kumar (2013) concluded that the main environmental risk factors include high calorie intake, physical inactivity, family history and genetic predisposition [35]. In their review, Pouwer et al. (2010) found that emotional stress and anxiety, sleep problems and the presence of stress and hostility were associated with an increased risk of developing T2DM [36]. Perceptions of illness and risk may have implications for improving health-promoting self-care in people with T2DM [37]. Individuals diagnosed with T2DM can influence the progression and development of their illness by participating in their own care [38].

The Brief Illness Perception Questionnaire has been validated already before, but not in the Slovenian language. A content validation of Brief Illness Perception Questionnaire was conducted to evaluate the questionnaire for use in patients with T2DM in Slovenia. Our calculations show a high degree of agreement among the evaluators on the relevance of the individual statements in the questionnaire. We also found that the kappa exceeded 0.74, indicating that the validity of the questions in the questionnaire is excellent. The questionnaire has already been evaluated in patients with T2DM [39,40] and other chronic diseases: heart failure [41], spinal cord injury [22], cancer patients [42,43,44], patients with chronic low back pain [45], tuberculosis [46] and many others [3]. Chew et al. (2017) found that the psychometric properties of each item were moderate, as was the test-retest reliability [39]. The Bahasa version of the Brief Illness Perception Questionnaire has been shown to be a valid and reliable scale for measuring illness perception in Indonesians with T2DM [40]. The internal consistency of the questionnaire was satisfactory (0.74) for the field questionnaire as measured by Cronbach’s alpha. Karatas et al. (2017), in their study in Turkey among cancer patients, found the questionnaire to be highly reliable for this target group, with a Cronbach alpha of 0.85 [42].

The limitations of this research are in the way the sample was obtained and the sampling method used. Due to the ad hoc sampling, where we selected participants according to their availability, there was also a possibility of bias in the selection of participants. To avoid bias in sample selection, we used both an online survey and a paper-based survey in clinics to collect data. This was done to avoid under-representation of certain target groups (e.g., those who do not use digital technology, those with more compromising perceptions).

## 5. Conclusions

Although T2DM is not perceived as very threatening by participants, there are still many areas for improvement. Also, the factors that patients consider important in the onset and progression of the illness show us the potential for incorporating these into health education work. The questionnaire was found to be a valid and reliable tool and should be used in the future in a larger population and over a longer longitudinal period to better assess the factors influencing the illness and the relationship between illness perception and self-management of T2DM.

## Figures and Tables

**Table 1:** Brief Illness Perception Questionnaire dimension [20].

	Male	Female	Total
Brief IPQ Dimension	Mean (SD)	Mean (SD)	Mean (SD)
Consequences	5.04 (2.81)	4.71 (2.78)	4.86 (2.78)
Timeline	8.96 (1.99)	9.04 (2.19)	9.02 (2.09)
Personal control	2.81 (1.82)	3.07 (1.85)	2.97 (1.83)
Treatment control	1.15 (1.64)	1.03 (1.67)	1.10 (1.65)
Identity	3.20 (2.78)	3.78 (2.76)	3.52 (2.77)
Coherence	2.37 (2.17)	2.00 (2.14)	2.17 (2.15)
Emotional representation	3.22 (3.08)	4.41 (3.24)	3.89 (3.20)
Concern	4.76 (3.09)	5.36 (3.24)	5.10 (3.17)
Total	31.52 (10.46)	32.97 (9.69)	32.37 (10.21)

**Table 2:** Linear regression of BIPQ total score.

	Unstandardized Coefficients		Standardized Coefficients	t	Sig.
	B	Std. Error	Beta		
(Constant)	46.442	7.945		5.846	<0.001
Age (years)	−0.280	0.132	−0.311	−2.125	0.038
Duration of disease (years)	0.202	0.094	0.308	2.142	0.036
Comorbidity (obesity)	0.377	2.691	0.018	0.140	0.889
Comorbidity (cardiovascular disease)	4.099	3.209	0.167	1.277	0.207

Dependent Variable: BIPQ Total Score.

**Table 3:** Content validity of the Brief Illness Perception Questionnaire [20].

No.	Question(s)	N	A	I-CVI	UA	Pc	k	Interpretation
1	How much does your illness affect your life?	6	5	0.833	0.000	0.094	0.816	Appropriate
2	How long do you think your illness will continue?	6	5	0.833	0.000	0.094	0.816	Appropriate
3	How much control do you feel you have over your illness?	6	5	0.833	0.000	0.094	0.816	Appropriate
4	How much do you think your treatment can help your illness?	6	6	1.000	1.000	0.016	1.000	Appropriate
5	How much do you experience symptoms from your illness?	6	6	1.000	1.000	0.016	1.000	Appropriate
6	How concerned are you about your illness?	6	5	0.833	0.000	0.094	0.816	Appropriate
7	How well do you feel you understand your illness?	6	6	1.000	1.000	0.016	1.000	Appropriate
8	How much does your illness affect you emotionally? (e.g., does it make you angry, scared, upset or depressed?)	6	5	0.833	0.000	0.094	0.816	Appropriate
9	Please list in rank-order the three most important factors that you believe caused your illness.	6	5	0.833	0.000	0.094	0.816	Appropriate

A = number of agreements; I-CVI = item content validity index; k = kappa designating agreement on relevance; No. = number of questions; N = sample size; Pc = probability of chance agreement, UA = universal agreement.

**Table 4:** Reliability analysis of the Brief Illness Perception Questionnaire [20].

Brief IPQ Dimension	Item-Total Correlation	Cronbach’s Alpha If Item Deleted
Consequences	0.555	0.456
Timeline	−0.084	0.645
Personal control	0.116	0.596
Treatment control	0.130	0.592
Identity	0.481	0.486
Coherence	−0.59	0.640
Emotional representation	0.627	0.411
Concern	0.462	0.487

## Data Availability

Data are available upon request from the corresponding author.
